# Quality characteristics and volatile profiles of giant salamander (*Andrias davidianus*) meat during the air frying process

**DOI:** 10.3389/fnut.2025.1649069

**Published:** 2026-01-16

**Authors:** Zhixin Luo, Kaiqi Cheng, Haicheng Li, Hui Yang, Yecheng Ran, Zhou Yang, Wen Su, Linjie Xi, Wengang Jin, A. M. Abd El-Aty, Ruichang Gao

**Affiliations:** 1 Qinling-Bashan Mountains Biological Resources Comprehensive Development 2011 C. I. C., Shaanxi University of Technology, Hanzhong, China; 2 Department of Pharmacology, Faculty of Veterinary Medicine, Cairo University, Giza, Egypt; 3 Department of Medical Pharmacology, Medical Faculty, Ataturk University, Erzurum, Türkiye; 4 School of Food and Biological Engineering, Jiangsu University, Zhenjiang, China

**Keywords:** giant salamander, air frying, headspace-gas chromatography–ion mobility spectrometry, volatile organic compounds, levels of relative odor activity

## Abstract

**Introduction:**

This study investigated the effects of air frying duration on the texture, color, and flavor characteristics of Andrias davidianus meat slices.

**Methods:**

Meat slices were air fried at 180 °C for 0–20 min. Quality attributes and volatile compounds were analyzed using HS-GC-IMS and multivariate statistical analysis.

**Results:**

With increasing frying durations, the shear force and a^*^ values significantly increased (*P* < 0.05), whereas the yield and L^*^ values decreased (*P* < 0.05), and the b^*^ value peaked at 15 min. A total of 48 volatile compounds, including aldehydes, ketones, esters, acids, alcohols, terpenes, and sulfides. The aldehyde and ketone contents increased with frying durations, whereas the ester and sulfide contents decreased, and the acid contents increased and then decreased. Principal component analysis (PCA) effectively distinguished samples at different frying stages, and orthogonal partial least squares discriminant analysis (OPLS-DA) identified 22 key volatiles with variable importance in projection (VIP) > 1. Relative odor activity value (ROAV) analysis further revealed 15 odor-active compounds (ROAVs = 1), among which 1-octen-3-ol, hexanal, ethyl 2-methylpropanoate, methyl benzoate, and diallyl disulfide were key aromatic substances.

**Conclusions:**

Air frying at 180 °C for 10–15 min resulted in more desirable quality and flavor characteristics, providing guidance for the development of ready-to-eat giant salamander meat products.

## 1 Introduction

The giant salamander (*Andrias davidianus*) belongs to the Cryptobranchidae family and is among the world's largest extant amphibians (1). As a rare species, the wild giant salamander is a National Grade II Protected Animal under current Chinese laws and regulations. Nevertheless, the offspring from captive breeding and farming can be processed and exploited as aquatic products or functional ingredients (2). In recent years, the giant salamander farming industry has developed rapidly in Shaanxi, Hunan, and Guizhou in China because of the maturation of artificial breeding techniques (3). The success of farming technology has made the market price of giant salamanders more affordable, which has provided a stable raw material base for the development of deep-processing products (4). Giant salamanders have attracted attention for their high nutritional and medicinal value (4). Its meat is rich in high-quality proteins, collagen, active peptides, and essential fatty acids, among other biologically active substances, and it has a variety of functional properties, such as antioxidant, antibacterial, and antiaging properties (1). With the development of giant salamander aquaculture and the emerging concept of “Great Health” (1, 5), how to increase the utilization of its resources and develop new food products through further processing technologies (e.g., air frying) has become a common concern for both industry and academia.

In modern cooking practices, meat is often processed by roasting, boiling, and frying (6). Frying is an important way to endow food with a unique flavor and texture. However, traditional frying methods may lead to oil oxidation and the generation of harmful substances. In recent years, air frying (AF), a low-fat, convenient, and good-flavor alternative, has gradually become favored by consumers. Compared with traditional frying, air frying achieves food heating through hot air circulation, significantly reducing the use of oil, and has obvious advantages in flavor preservation and nutrient retention (7, 8). In the process of air frying, lipids, proteins, and carbohydrates in food undergo a thermal degradation reaction (9) and then generate a wide variety of volatile organic compounds (VOCs), which directly affect flavor shaping, aroma emission, and the overall sensory quality of the finished product (10). Therefore, accurate analysis and identification of the composition of and changes in VOCs are highly important for optimizing processing technology and improving product quality.

Headspace-gas chromatography–ion mobility spectrometry (HS-GC–IMS) combines the high separation capability of gas chromatography (GC) with the rapid response of ion mobility spectrometry (IMS). This technique enables high-sensitivity detection at atmospheric pressure without complex pretreatment, offering a short analysis time and low detection limits (11–13). Compared with conventional gas chromatography–mass spectrometry (GC–MS), HS-GC–IMS has superior sensitivity for low-concentration volatile compounds and generates two- or three-dimensional volatile fingerprint spectra, allowing rapid visual differentiation of sample flavors (12, 13). While electronic noses provide simple operation and real-time analysis, they are limited in compound-level identification. In contrast, HS-GC–IMS captures the overall odor profile and identifies key volatile components, providing a more comprehensive perspective for food flavor analysis and sample characterization (11–13). At present, HS-GC–IMS technology is frequently employed in food analysis, biomedical and clinical detection, environmental detection, and other fields (14–16). In the field of aquatic products, this technology has formed a mature application system. For example, Liu et al. (17) analyzed precursor substances via HPLC and evaluated flavor changes via HS-GC–IMS, electronic tongue, and electronic nose techniques, systematically revealing the dynamic influence of different processing steps on the flavor of beer. Han et al. (18) utilized chemical analysis methods such as HS-GC–IMS in conjunction with sensory evaluation to analyze the similarities and variations in the taste and odor of squid from various habitats. Li et al. (19) systematically analyzed the changes and dynamic characteristics of volatile flavor compounds during the fermentation of traditional Chinese shrimp paste by integrating electronic nose, solid-phase microextraction-gas chromatography-mass spectrometry, and HS-GC–IMS techniques. These studies demonstrated that HS-GC–IMS technology can be employed to determine the flavor change profile of giant salamander meat during different frying durations, thus providing strong support for scientifically and reasonably optimizing cooking conditions, improving flavor, and ensuring food safety.

Our previous study characterized the quality traits and flavor volatiles of giant salamander meatballs fried via different methods (20). Given the popularity of air-fried ready-to-eat products, as well as the development demand for giant salamander value-added products, it is necessary to investigate the quality characteristics and flavor profiles of air-fried giant salamander meat products. However, few reports in this regard can be found. Therefore, the objectives of this research were to investigate changes in the yield, shear force, color values, and sensory evaluations of *Andrias davidianus* meat during air frying (180 °C, 0–20 min). Moreover, the VOCs present in *Andrias davidianus* meat during air frying were also characterized via HS-GC–IMS and chemometrics, which could provide a foundation for optimizing processing technology and improving product flavor quality in the future.

## 2 Materials and methods

### 2.1 Materials and reagents

Fresh and live giant salamanders (5 tails, 2.25 ± 0.25 kg) were acquired from Longtoushan Aquaculture Co., Ltd. (Hanzhong, China), slaughtered, cleaned, eviscerated, vacuum packaged, and transported to the laboratory under frozen conditions.

Cooking wine was purchased from Yangxi Meiweixian Seasoning Food Co., Ltd. (Guangdong, China). Table salt was purchased from Sichuan Jiuda Penglai Salt Co., Ltd. (Chengdu, China). Edible rapeseed oil was purchased from YIHAI KERRY Food Industry Co., Ltd. (Xianyang, China). Garlic and ginger were purchased from Lianhu Farmers' Market (Hanzhong, China).

The n-ketone standards (2-butanone, 2-pentanone, 2-hexanone, 2-heptanone, 2-octanone, and 2-nonanone) were analytically pure compounds purchased from Aladdin Company (Shanghai, China).

### 2.2 Sample preparation

The frozen giant salamander was first thawed and sliced into uniformly sized meat slices. The samples were subsequently marinated with ginger (1.0%, w/w), garlic (1.0%, w/w), cooking wine (2.0%, w/w), and salt (1.5%, w/w) to diminish the fishy odor. The formulation was established on the basis of previous studies on flavor optimization in seafood and fried products (21, 22) and further refined through preliminary sensory evaluation. After marination for 10 min, the ginger and garlic were removed, and the meat slices on the outside were equally covered with a thin coating of rapeseed oil. The sample was positioned in an air fryer and cooked for 0, 5, 10, 15, or 20 min at 180 °C. Following frying, the quality indicators were measured after the extra surface oil was removed with tissue paper.

### 2.3 Yield estimation

Referring to the experimental methods of Jin et al. (3) and Kim et al. (23) and modifying them, three meat slice samples from each treatment group with different durations of air frying (5, 10, 15, and 20 min) were weighed before and after frying. The percentage of mass loss at each time point was calculated via the following formula:


$$\begin{array}{l} Yield\left(\frac{g}{100g}\right)=\frac{sample\;weight\;after\;frying\;\left(g\right)}{sample\;weight\;before\;frying\;\left(g\right)}\times 100 \end{array}$$

### 2.4 Measurement of shear force

A digital screen muscle tenderness instrument (C-LM3B, Northeast China) was used to calculate the samples' shear force data at each air frying time (0, 5, 10, 15, and 20 min), and the greatest amount of force needed to shear each sample cross-section was recorded. Each group of samples had three measurements, and an analysis was conducted using the mean value.

### 2.5 Color value measurement

A color difference meter (SMY-200 series, Beijing Shengmingyang Science and Technology Development Co., Ltd.) was used to gauge the hue of the surface of each sample. Before measurement, a white calibration plate was used for standardization, and the values of L^*^ (brightness), a^*^ (red–green), and b^*^ (yellow–blue) were recorded. Each group has 4 samples, and it is determined that the characteristic hue is the average value.

### 2.6 Sensory evaluation

Sensory evaluation was conducted according to the requirements of the Chinese recommended national standard GB/T 22210−2008, with appropriate adjustments. The sensory evaluation was carried out by an evaluation panel consisting of 10 rigorously selected food science postgraduate students (5 male, 5 female, aged 18–28). All members met the following criteria: no taste or smell impairments, nonsmokers, no history of food allergies, and completed systematic training in the course “Food Sensory Evaluation” at Shaanxi University of Technology (including theoretical examinations and practical tests), enabling them to accurately identify the five basic tastes (sour, sweet, bitter, salty, and umami). The formal experiment was conducted in a standardized sensory laboratory at Shaanxi University of Technology (temperature: 25 ± 1 °C, uniform lighting, no interfering odors). To avoid order effects, a completely randomized block design was used, with samples marked with three-digit random codes for blind testing (24). Each evaluator independently conducted three repeated evaluations of five groups of samples with different frying durations, with a 2-min interval between each evaluation (during which the mouth was rinsed with warm water to remove residual flavors) (25). The evaluation indices include color, smell, texture, taste, and overall acceptability (26), which are rated on a nine-point liking scale (1 for extremely disliked and 9 for extremely liked) (23), and the ultimate score is calculated by taking the mean of every item's values.

### 2.7 HS-GC–IMS detection of VOCs

The HS-GC–IMS assay was performed according to the methods of Li et al. (19) with some modifications. A 20.0 ml headspace vial was filled with a precisely weighed 1.0 g portion of giant salamander meat. For every sample, three parallel example groups were created (19, 20). To encourage gas release, the components were oscillated at 500 r/min for 20 min while cultured at 60 °C. Following incubation, 500.0 μl of gas sample was drawn from the headspace with an airtight injection needle and injected into a Flavorspec^®^ GC–IMS (G.A.S., Germany) for GC–IMS analysis. The headspace injection conditions were splitless mode, and the injection needle temperature was set at 85 °C. The gas chromatography conditions were as follows: the carrier gas was nitrogen with a purity of 99.999%, the column temperature was 60 °C, and the initial carrier gas flux rate was 2.00 mL/min. After 2 min, the linearity increased to 10.00 ml/min in 8 min and then to 100.00 ml/min in 10 min. Finally, the mixture was incubated for 5 min. The total operation time of chromatography was 25 min. The IMS detection parameters included the following: ionization source, tritium (3H); migration tube length, 53 mm; electric field intensity, 500 V/cm; migration tube temperature, 45 °C; drift gas, high-purity nitrogen (purity ≥ 99.999%); flow rate, 150 ml/min; and positive ion detection mode, 45 °C. A mixed standard of external standard substances (n-ketones C4–C9) was detected, calibration curves for retention time (RT) and retention index (RI) were established, and then the RI of the target substance was calculated on the basis of its RT. The built-in GC RI (NIST 2020) database and IMS detection time (DT) database in VOCal software were used for retrieval and comparison to perform qualitative analysis of the target substance (20, 27).

### 2.8 Relative odor activity value

The relative odor activity value (ROAV) method was used to screen and identify the primary volatile flavor substances of the samples at various frying stages (28). Referring to the method of Li et al. (19), the ROAV value of the component that contributes the most to the samples' overall flavor was defined as 100, and the ROAV values of the other components were computed as ROAV via the following equation:


$$\begin{array}{l} ROAV\approx \frac{C_{i}}{C_{s}}\times \frac{T_{s}}{T_{i}}\times 100 \end{array}$$

where *C*_*S*_ and *T*_*S*_ represent the relative content and odor threshold of the volatile substances that contribute the most to the sample's flavor, respectively, and *C*_*i*_ and *T*_*i*_ represent the relative content and odor threshold of each volatile ingredient in the sample, respectively.

### 2.9 Data processing

Using plugins such as Reporter and Gallery Plot in the VOCal data processing software, three-dimensional spectra, two-dimensional spectra, difference spectra, and fingerprints of volatile components were generated. Aromatic compounds were identified on the basis of the NIST 2020 and IMS databases. The relative amounts of various volatile components were computed by normalizing the peak volumes. The data are expressed as the average ± standard deviation (*n* ≥ 3) and were analyzed via one-way analysis of variance (ANOVA) with SPSS 27.0.1 (*P* < 0.05). Plots were generated via Origin 2021 software and online websites (https://cloud.metware.cn/#/home and https://www.chiplot.online/circle_heatmap.html).

## 3 Results and discussion

### 3.1 Quality attributes of giant salamander meat after varying durations of AF

**Figure 1:**
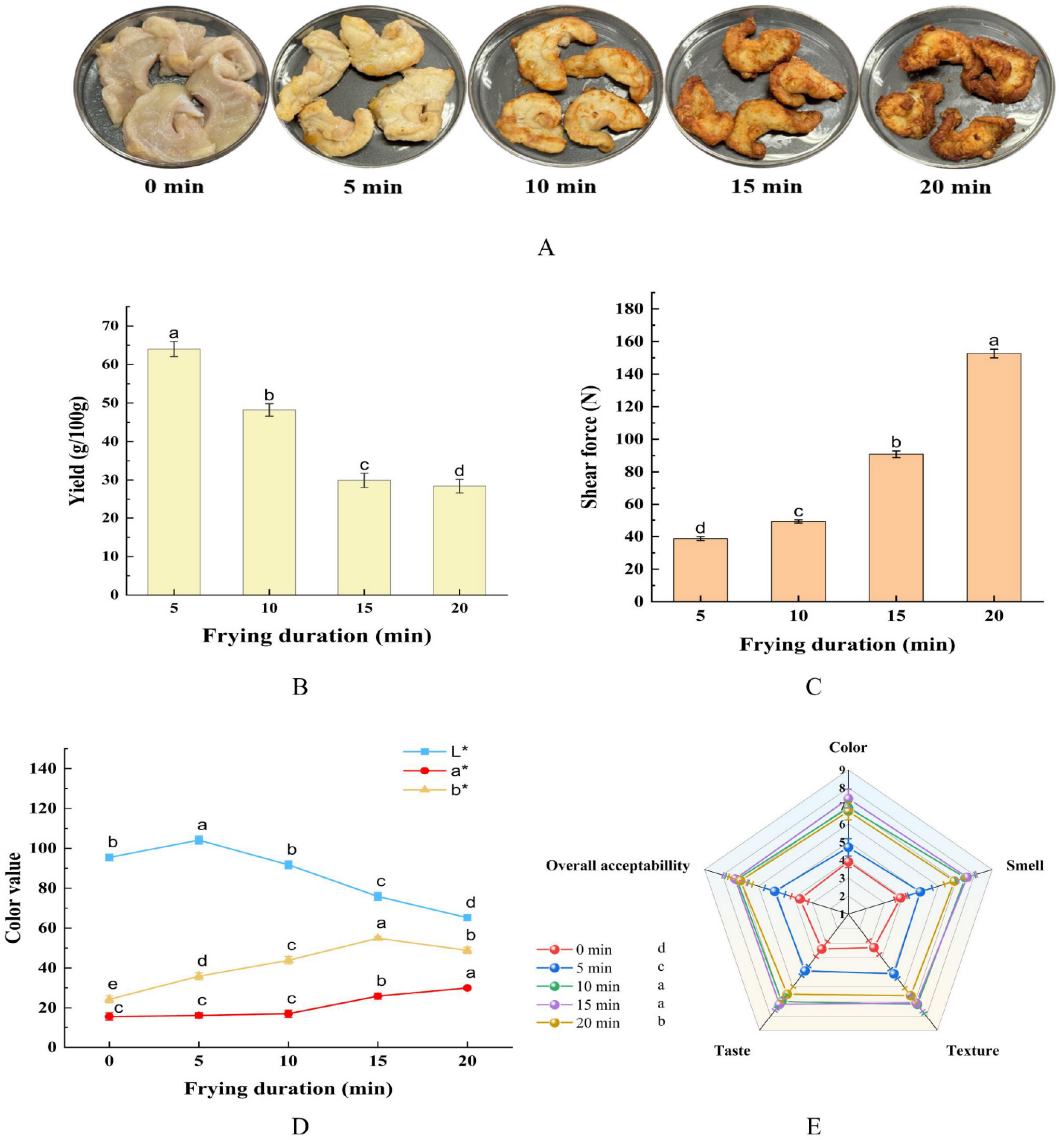
Effects of different AF durations on the meat quality attributes of giant salamanders: **(A)** photograph of their appearance; **(B)** processing yield; **(C)** shear force; **(D)** color value; **(E)** sensory evaluation. Data in the figure are expressed as average ± standard deviation (*n* ≥ 3) and were analyzed by one-way ANOVA using SPSS 27.0.1. Different lowercase letters indicate significant differences (*P* < 0.05).

The processing quality characteristics of meat products mainly include yield, color, texture, and other indicators. In this research, the effects of the air frying durations on the processing yield, shear force, color value (L^*^, a^*^, b^*^), and sensory evaluation of giant salamander meat slices were measured. The results are shown in Figures 1A–E.

Figure 1A shows images of giant salamander meat slices after different durations of air frying. The experimental observations revealed that with increasing air frying durations, the surface of the giant salamander meat samples gradually presented an obvious curling phenomenon, and the degree of curling increased with increasing heat treatment time. In addition, the color of the sample gradually changed from the initial raw meat state to a burnt yellow color. When the treatment time reaches 20 min, a local coking phenomenon appears at the edge of the sample, which indicates that the time parameter has approached or exceeded the optimal heat treatment threshold of the sample.

Figure 1B shows that the sample processing yield decreased dramatically as the air frying durations increased (*P* < 0.05), with values of 64.01% in the 5-min group and 48.20% in the 10-min group. The yield of the 15-min treatment group decreased sharply to 29.92%, and that of the 20-min group tended to be stable at 28.40%. This phenomenon is due mainly to the continuous evaporation loss of water during high-temperature processes. Similar phenomena were also reported in the studies of Li et al. (29) and Lin et al. (30).

The shear force of the giant salamander meat samples subjected to different durations of air frying significantly increased nonlinearly (*P* < 0.05) (Figure 1C), increasing from 38.67 N in 5 min to 152.67 N in 20 min, especially in the range of 15–20 min. This change may be due to heat denaturation of myofibrillar protein at elevated temperatures, which leads to enhanced cross-linking between the fibers and protein, and at the same time, the massive loss of water jointly aggravates the texture hardening process (31–33).

The color value change trend is shown in Figure 1D. The L^*^ value of the air-fried meat slices increased significantly during the initial 0–5 min (*P* < 0.05), which was presumed to be due to the changes in muscle microstructure caused by protein thermal denaturation, which increased light reflection (34, 35), and then continued to decrease for 5–20 min, resulting in the surface darkening of the meat slices, which was attributed mainly to the synergistic effect of the Maillard reaction and caramelization (36). The a^*^ value generally tended to increase (*P* < 0.05), with a very notable increase from 10–15 min, which may be related to the pigment concentration effect caused by the massive production of reddish-brown products in the middle of the Maillard reaction and the complete evaporation of surface water. The increase in the b^*^ value at 0–15 min reflects the accumulation of yellow intermediate products in the early stage of Maillard, whereas the decrease at 15–20 min may be due to the masking effect of dark melanoids produced by excessive browning of yellow tones. According to the variation characteristics of each parameter, the a^*^ and b^*^ values reached a high level synchronously in the 10–15 min period. At this time, the meat slices presented an ideal red and yellow composite color, which was the best color development stage of the air frying process.

Sensory evaluation results (Figure 1E) revealed that during air-frying, the overall sensory quality of *Andrias davidianus* meat slices first increased but then decreased with prolonged heating. At 0 min (raw sample), all the sensory scores were relatively low (color 3.9, smell 3.9, texture 3.3, taste 3.4, and overall acceptability 3.7), reflecting the unprocessed state of raw meat with undeveloped flavor and texture. After 5 min of air-frying, both color and aroma improved markedly (approximately 5.0), and the texture and taste were enhanced, indicating that short-term heating began to generate attractive sensory characteristics. At 10 min, all attributes increased significantly (approximately 6–7 points), as the Maillard reaction and caramelization contributed to a richer flavor and a tender-crisp texture, resulting in a noticeable increase in overall acceptability. The highest sensory scores were observed at 15 min (color 7.4, smell 7.6, texture 7.1, taste 7.2, overall acceptability 7.3), characterized by a golden color, full aroma, and a crispy yet juicy texture. At 20 min, some attributes slightly declined (color 6.7, texture 6.6, taste 6.5), possibly due to mild bitterness or hardness caused by overfrying, although smell (6.9) and overall acceptability (7.0) remained at relatively high levels. Overall, the optimal sensory quality of air-fried *Andrias davidianus* meat slices was achieved between 10 and 15 min, whereas longer frying durations may lead to quality deterioration. The sensory evaluation was conducted by trained panelists through repeated tests, ensuring good consistency and reliability (20, 23). Future studies could expand the sample size and incorporate consumer testing to further validate product acceptability.

### 3.2 3D and 2D spectra of VOCs from AF giant salamander meat at different durations via HS-GC–IMS

**Figure 2:**
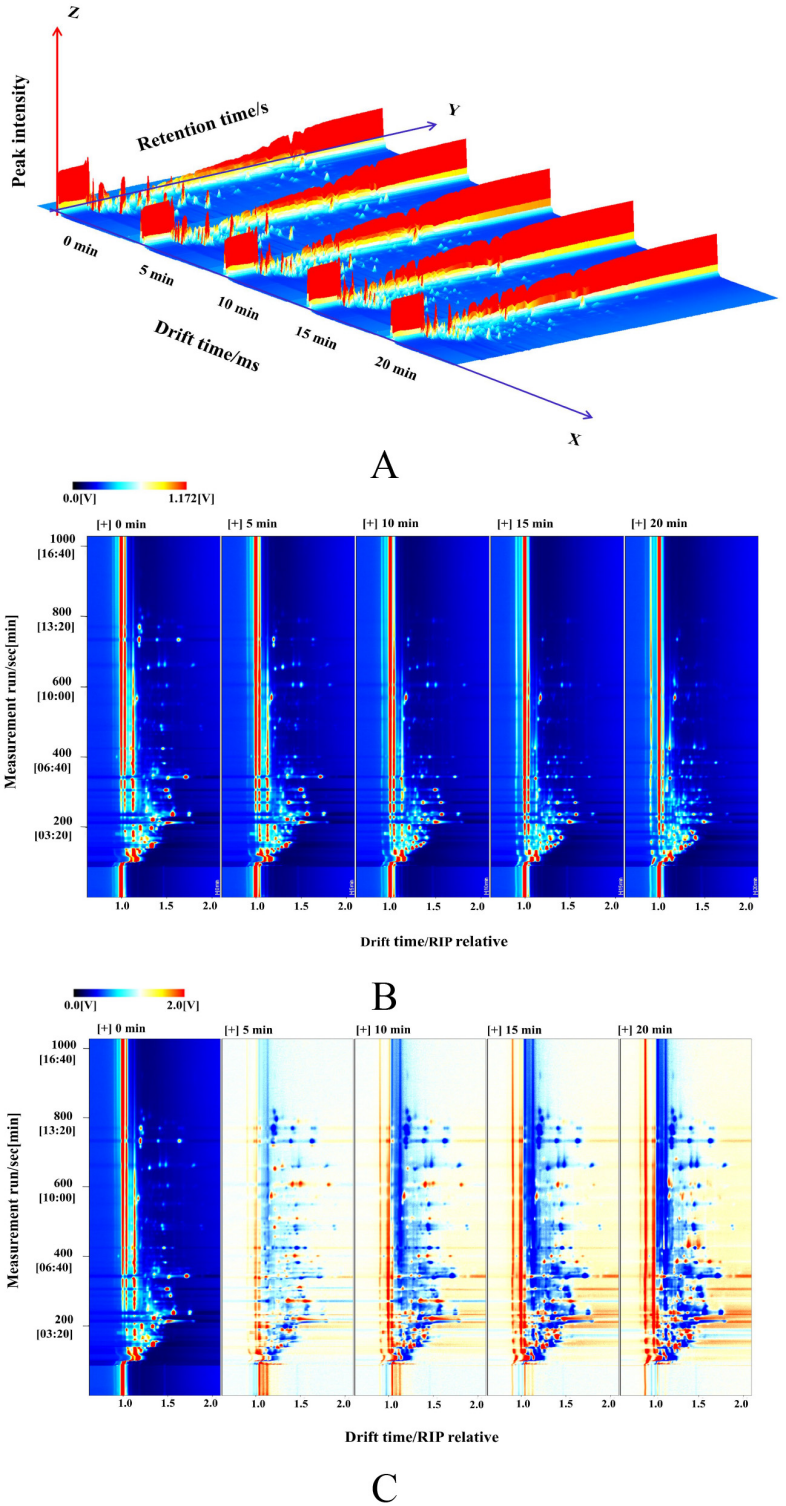
HS-GC–IMS spectra of giant salamander meat with different frying durations. **(A)** 3D map; **(B)** 2D map; **(C)** 2D difference spectrum.

HS-GC–IMS technology was employed to identify volatile organic compounds (VOCs) in giant salamander meat after five different durations of air frying (0, 5, 10, 15, and 20 min). Figure 2A shows the distribution of VOCs in the samples at each time point. The order of the sample arrangement from left to right corresponds to the different durations of air frying. The seat axes represent the removal time (*X*-axis), reservation time (*Y*-axis), and semaphore crown intensity (*Z*-axis), and the color gradient reflects the difference in ion intensity (37).

The three-dimensional map is projected to the retention time migration time plane to obtain a two-dimensional dot matrix map (Figure 2B). Taking the 0-min non-air-fried sample as the benchmark, the spectrum of the additional samples was subtracted and analyzed to acquire a disparity map (Figure 2C), which makes it relatively easy to contradistinguish the subtle variations in volatile substances between the meat of giant salamanders with different durations of air frying. In the comparison diagram, the hue represents the strength of the volatile chemical signal, the white area shows that the density of the object sample is consistent with that of the reference sample, the blue region indicates that the concentration of the target sample is weaker than that of the reference, and the red area indicates that the density is higher than that of the contrast. The figure shows that with increasing duration of air frying, the kinds and relative components of VOCs in the meat samples from the giant salamander significantly changed dynamically, indicating that HS-GC–IMS technology can separate VOCs effectively in various periods.

### 3.3 Fingerprint analysis of VOCs in giant salamander meat at different AF durations

The retention indices of the volatile compounds were estimated by constructing a calibration curve with external standards (n-ketones C4–C9) on the basis of their retention times and corresponding retention indices. The qualitative analysis of volatile compounds was subsequently performed by matching the experimentally determined retention index and detection time (IMS) against the built-in databases of the VOCal software (27). A total of 51 signal peaks (comprising monomers and dimers) were discovered in five samples at different durations, and 48 VOCs were determined, encompassing 13 aldehydes, 11 alcohols, 10 esters, 6 ketones, 3 acids, 3 terpenes, and 2 sulfides.

The retention index (RI), retention time (RT), detection time (DT), and relative content of each VOC are listed in Table 1. The greatest increase was found in butanone overall, followed by acetic acid D, ethyl acetate D, isopentyl alcohol D, and acetic acid M. In terms of flavor dynamic evolution, different VOCs exhibited widely disparate patterns. The alcohols n-hexanol D and (E) 3-hexen-1-ol D, for example, decayed progressively with time, although isopentyl alcohol and hexanal tended to accumulate continuously.

**Table 1:** Volatile organic compounds identified in giant salamander meat.

**Count**	**Classification**	**Index**	**RI**	**Rt [sec]**	**Dt [a.u.]**	**Relative content (%)**				
						**0 min**	**5 min**	**10 min**	**15 min**	**20 min**
1	Acids	Hexanoic acid	1,041.8	655.468	1.2938	1.60 ± 0.03^b^	1.80 ± 0.05^a^	1.04 ± 0.02^c^	0.60 ± 0.03^d^	0.47 ± 0.02^e^
2		Acetic acid D	599.8	131.913	1.04882	8.38 ± 0.21^a^	8.63 ± 0.96^a^	10.73 ± 2.24^a^	12.41 ± 0.90^a^	11.32 ± 4.39^a^
3		Acetic acid M	587.7	126.793	1.13462	3.13 ± 0.14^b^	4.75 ± 0.50^a^	6.13 ± 0.63^a^	5.79 ± 0.35^a^	4.98 ± 1.62^a^
4	Alcohols	1-Octen-3-ol	988.9	556.606	1.16067	0.42 ± 0.02^b^	0.43 ± 0.08^b^	0.63 ± 0.03^a^	0.51 ± 0.02^b^	0.32 ± 0.04^c^
5		n-Hexanol D	872.4	358.213	1.63851	0.33 ± 0.03^a^	0.26 ± 0.06^b^	0.16 ± 0.00^c^	0.11 ± 0.01^c^	0.10 ± 0.02^c^
6		n-Hexanol M	873.7	359.913	1.32748	0.68 ± 0.02^b^	0.75 ± 0.04^a^	0.62 ± 0.01^c^	0.55 ± 0.02^d^	0.40 ± 0.03^e^
7		(E)-3-hexen-1-ol M	863.3	346.316	1.25003	0.41 ± 0.04^d^	0.83 ± 0.07^c^	0.97 ± 0.04^b^	1.15 ± 0.02^a^	0.86 ± 0.11^bc^
8		(E)-3-hexen-1-ol D	862.8	345.75	1.52417	1.18 ± 0.11^a^	0.95 ± 0.04^b^	0.58 ± 0.02^c^	0.45 ± 0.02^d^	0.21 ± 0.02^e^
9		2-Ethyl-1-hexanol M	1,045.9	663.563	1.41923	0.66 ± 0.11^a^	0.56 ± 0.15^ab^	0.42 ± 0.09^bc^	0.32 ± 0.04^cd^	0.17 ± 0.00^d^
10		2-Ethyl-1-hexanol D	1,045.9	663.563	1.79567	0.46 ± 0.09^a^	0.24 ± 0.08^b^	0.10 ± 0.01^c^	0.07 ± 0.01^c^	0.07 ± 0.01^c^
11		Benzyl alcohol	1,016.5	607.977	1.50134	0.54 ± 0.04^c^	1.17 ± 0.03^a^	1.05 ± 0.09^b^	0.45 ± 0.02^c^	0.18 ± 0.01^d^
12		2,3-Butanediol	795.4	269.345	1.37289	1.57 ± 0.07^e^	3.87 ± 0.08^b^	4.16 ± 0.10^a^	3.14 ± 0.06^c^	2.24 ± 0.17^d^
13		Isopentyl alcohol D	740.1	216.606	1.49674	4.82 ± 0.12^c^	5.76 ± 0.23^b^	6.75 ± 0.16^a^	6.68 ± 0.13^a^	5.16 ± 0.28^c^
14		Isopentyl alcohol M	741.6	217.876	1.24963	0.56 ± 0.00^d^	0.70 ± 0.10^d^	1.30 ± 0.11^c^	2.19 ± 0.04^b^	2.92 ± 0.52^a^
15	Aldehydes	Octanal M	1,017.9	610.554	1.41349	0.39 ± 0.02^e^	1.15 ± 0.04^c^	1.67 ± 0.08^a^	1.31 ± 0.04^b^	0.77 ± 0.06^d^
16		2-Heptenal (E)	963.3	505.08	1.26099	0.37 ± 0.00^d^	0.53 ± 0.02^b^	0.59 ± 0.05^a^	0.55 ± 0.00^ab^	0.47 ± 0.03^c^
17		Heptanal D	903.5	402.403	1.69137	0.07 ± 0.01^d^	0.15 ± 0.01^b^	0.26 ± 0.00^a^	0.16 ± 0.01^b^	0.10 ± 0.02^c^
18		Heptanal M	902.8	401.27	1.34346	0.18 ± 0.01^d^	0.37 ± 0.02^c^	0.66 ± 0.02^a^	0.70 ± 0.05^a^	0.55 ± 0.04^b^
19		Hexanal M	797.8	271.712	1.26631	0.74 ± 0.02^d^	0.94 ± 0.05^c^	1.17 ± 0.04^b^	1.63 ± 0.04^a^	1.73 ± 0.18^a^
20		Hexanal D	797.8	271.712	1.55518	0.31 ± 0.02^e^	1.82 ± 0.08^c^	2.73 ± 0.08^a^	2.05 ± 0.14^b^	0.75 ± 0.04^d^
21		2-Methylbutanal M	685.6	174.541	1.17929	0.23 ± 0.01^c^	0.18 ± 0.01^c^	0.62 ± 0.07^b^	1.13 ± 0.02^a^	1.35 ± 0.37^a^
22		2-Methylbutanal D	682.4	172.732	1.40037	0.36 ± 0.04^bc^	0.25 ± 0.01^c^	1.11 ± 0.06^b^	2.72 ± 0.04^a^	2.79 ± 0.93^a^
23		3-Methylbutanal M	668.3	164.977	1.19467	0.29 ± 0.03^b^	0.31 ± 0.05^b^	0.29 ± 0.08^b^	0.51 ± 0.04^ab^	0.76 ± 0.44^a^
24		3-Methylbutanal D	671.4	166.622	1.4057	0.32 ± 0.04^d^	0.19 ± 0.02^d^	0.95 ± 0.06^c^	2.15 ± 0.04^b^	2.74 ± 0.35^a^
25		Octanal D	1,015.5	606.24	1.82094	0.08 ± 0.01^d^	0.37 ± 0.01^b^	0.49 ± 0.07^a^	0.18 ± 0.01^c^	0.08 ± 0.01^d^
26		Benzaldehyde	964.9	508.097	1.46723	0.09 ± 0.01^d^	0.19 ± 0.02^bc^	0.24 ± 0.02^a^	0.20 ± 0.01^b^	0.17 ± 0.01^c^
27		2-Furfural	832.5	308.982	1.09346	0.76 ± 0.02^b^	0.70 ± 0.02^bc^	0.59 ± 0.03^c^	0.71 ± 0.01^bc^	2.41 ± 0.17^a^
28	Esters	Ethyl 2-methyl propanoate D	763.4	237.73	1.57329	6.89 ± 0.08^a^	5.47 ± 0.15^b^	4.27 ± 0.15^c^	3.33 ± 0.04^d^	1.69 ± 0.52^e^
29		Ethyl 2-methyl propanoate M	760.2	234.789	1.1956	0.17 ± 0.01^d^	0.30 ± 0.02^cd^	0.52 ± 0.06^c^	1.09 ± 0.01^b^	1.82 ± 0.32^a^
30		Ethyl acetate D	631.8	146.442	1.32882	17.20 ± 0.33^a^	13.90 ± 0.46^b^	8.40 ± 0.82^c^	6.13 ± 0.34^d^	4.00 ± 1.87^e^
31		Ethyl acetate M	634.3	147.616	1.10142	0.11 ± 0.01^d^	0.19 ± 0.00^c^	0.27 ± 0.02^b^	0.36 ± 0.02^a^	0.35 ± 0.01^a^
32		Ethyl levulinate M	1,074.1	721.47	1.19877	1.96 ± 0.13^a^	1.69 ± 0.06^b^	0.68 ± 0.05^c^	0.31 ± 0.07^d^	0.25 ± 0.02^d^
33		Ethyl levulinate D	1,073.2	719.559	1.63692	0.14 ± 0.03^b^	0.18 ± 0.02^a^	0.06 ± 0.01^c^	0.06 ± 0.00^c^	0.07 ± 0.00^c^
34		Isobutyl propionate	862.1	344.858	1.71691	4.78 ± 0.21^a^	3.33 ± 0.06^b^	1.51 ± 0.15^c^	0.64 ± 0.02^d^	0.20 ± 0.05^e^
35		Ethyl 2-hydroxy propanoate	813	287.512	1.53712	2.33 ± 0.07^a^	1.68 ± 0.14^b^	1.62 ± 0.17^b^	0.85 ± 0.06^c^	0.67 ± 0.09^c^
36		Methyl benzoate	1,096.4	770.956	1.20348	2.96 ± 0.10^a^	0.21 ± 0.01^b^	0.13 ± 0.01^b^	0.14 ± 0.01^b^	0.17 ± 0.03^b^
37		Methyl 2-furoate	966	510.266	1.15122	2.07 ± 0.09^a^	1.48 ± 0.04^b^	1.33 ± 0.03^c^	1.01 ± 0.01^d^	0.79 ± 0.03^e^
38	Ketones	2-Heptanone D	889.8	382.008	1.62621	0.05 ± 0.00^c^	0.11 ± 0.01^c^	0.11 ± 0.01^c^	0.39 ± 0.01^b^	0.75 ± 0.23^a^
39		2-Heptanone M	892.9	386.54	1.26232	0.15 ± 0.01^e^	0.27 ± 0.01^d^	0.37 ± 0.02^c^	1.04 ± 0.02^b^	1.65 ± 0.04^a^
40		Butanone	534.9	106.716	1.07793	10.02 ± 0.14^d^	13.88 ± 0.25^c^	17.03 ± 0.86^b^	20.34 ± 0.66^a^	20.33 ± 3.31^a^
41		6-Methyl-5-hepten-2-one	994.1	567.75	1.17835	2.34 ± 0.06^c^	3.79 ± 0.21^b^	5.45 ± 0.15^a^	5.16 ± 0.07^a^	4.04 ± 0.43^b^
42		Hexan-2-one	832.5	308.982	1.49501	5.91 ± 0.18^b^	6.59 ± 0.19^a^	4.51 ± 0.14^c^	1.35 ± 0.07^d^	0.54 ± 0.04^e^
43		2-Acetylfuran	919.2	427.031	1.11272	1.67 ± 0.13^b^	1.17 ± 0.09^c^	0.91 ± 0.04^d^	1.22 ± 0.02^c^	2.63 ± 0.14^a^
44	Terpenoids	Pinene	977.7	533.353	1.21473	0.43 ± 0.03^a^	0.40 ± 0.03^a^	0.26 ± 0.03^b^	0.18 ± 0.02^c^	0.15 ± 0.02^c^
45		Camphene	950.6	481.265	1.21314	0.50 ± 0.02^c^	0.95 ± 0.03^a^	0.65 ± 0.13^b^	0.36 ± 0.03^d^	0.30 ± 0.06^d^
46		Limonene	1,040.7	653.34	1.22109	0.43 ± 0.04^c^	1.05 ± 0.05^a^	0.82 ± 0.17^b^	0.35 ± 0.05^c^	0.32 ± 0.04^c^
47	Sulfides	Diallyl disulfide M	1,081.1	736.763	1.20248	3.85 ± 0.12^a^	2.10 ± 0.18^b^	0.95 ± 0.07^c^	0.81 ± 0.21^c^	0.69 ± 0.23^c^
48		Diallyl disulfide D	1,080.7	735.807	1.63568	1.74 ± 0.25^a^	0.33 ± 0.06^b^	0.08 ± 0.01^c^	0.07 ± 0.02^c^	0.06 ± 0.01^c^

The suffix D in the table represents dimers of volatile organic compounds, whereas M represents monomers. Data are expressed as average ± standard deviation (*n* = 3) and were analyzed by one-way ANOVA using SPSS 27.0.1. Different lowercase letters with superscripts in the same row denote significant differences between samples (*p* < 0.05).

**Figure 3:**
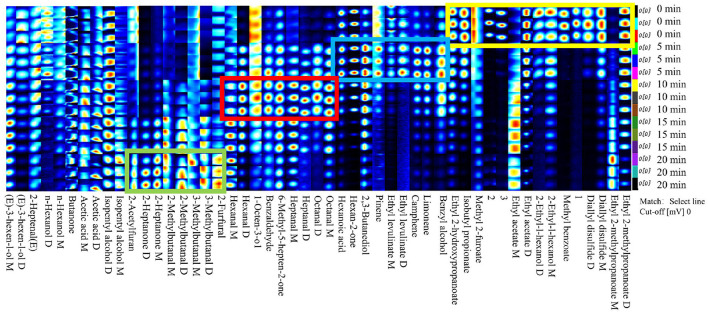
Fingerprints of volatile organic compounds of giant salamander meat with different durations of air frying by HS-GC–IMS: the vertical axis of the map represents the air frying durations (from top to bottom: 0 min, 5 min, 10 min, 15 min, and 20 min), and the horizontal axis corresponds to specific VOCs. Some substances are marked with D and M, representing monomers and dimers of the same compound, and unidentified peaks are represented by numbers.

Through spatiotemporal visualization analysis of the HS-GC–IMS fingerprints (Figure 3), the five groups of samples presented characteristic flavor evolution tracks during the 0–20 min air frying process. The contents of diallyl disulfide (D and M), 2-ethyl-1-hexanol (D and M), and a variety of esters (ethyl 2-methyl propanoate (D and M), methyl 2-fluoroate, ethyl acetate (D and M), isobutyl propionate, ethyl 2-hydroxy propanoate, and methyl benzoate) were relatively high in the 0 min sample (yellow box). The contents of ethyl levulinate (D and M), benzyl alcohol, 2,3-butanediol, hexan-2-one, hexanoic acid, and terpenoids (limonene, camphene, and pinene) were relatively high in the 5-min treatment group (blue box). After 10 min (red box), the samples entered the aldehyde ketone-dominant stage, and the contents of octanal (D and M), heptanal (D and M), benzaldehyde, 6-methyl-5-hepten-2-one, 1-octen-3-ol, and hexanal (D and M) were high. The green box samples, which were treated for 20 min, presented elevated levels of 2-furfural, 2-acetylfuran, 3-methylbutanal (D and M), 2-methylbutanal (D and M), and 2-heptanone (D and M). These giant salamander meats, which have different durations of air frying, have different flavor characteristic substances, which may affect the overall volatile flavor characteristics associated with various durations of frying.

### 3.4 Qualitative analysis of VOCs in giant salamander meat with different durations of AF

**Figure 4:**
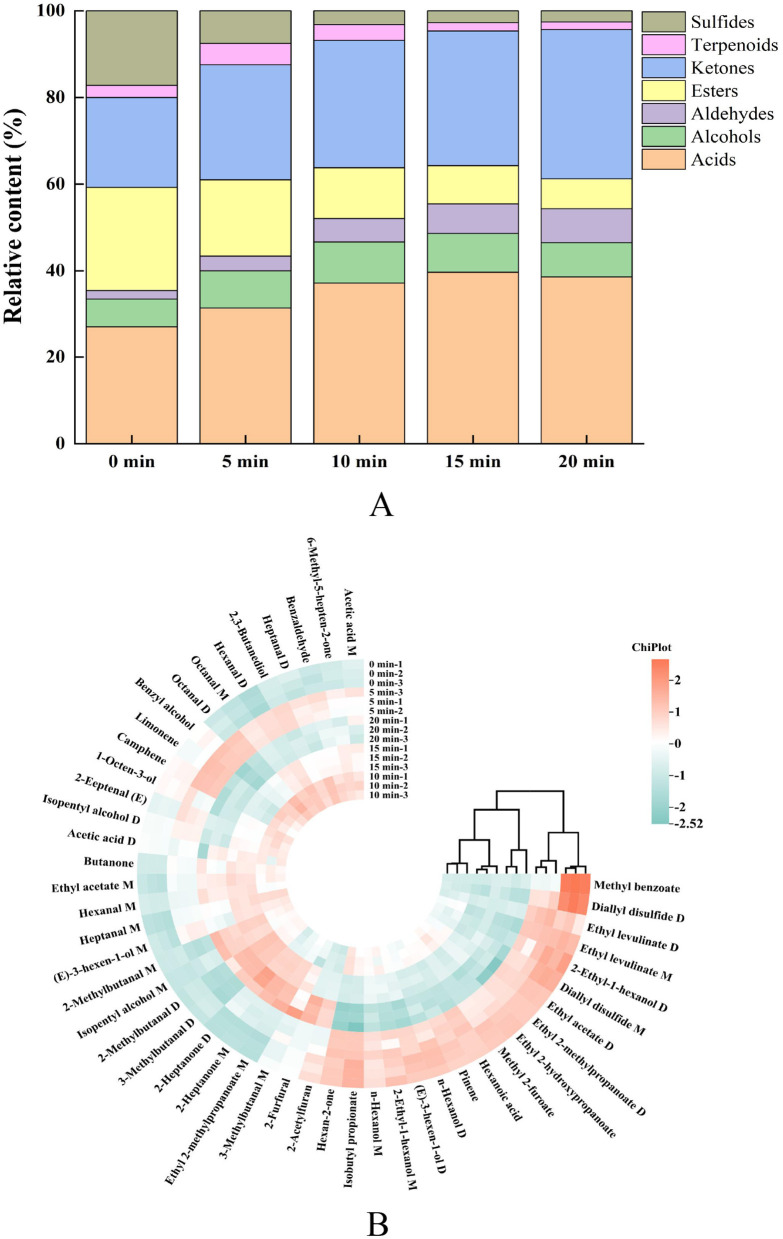
Dynamic changes in volatile flavor compounds of Chinese giant salamander meat during air frying: **(A)** relative content of volatile components; **(B)** cluster heatmap of flavor compounds.

To clearly illustrate the evolution of volatile components in *Andrias davidianus* meat during air-frying, the fingerprinting signal intensities were normalized by peak area, and the relative contents of various volatile compounds were visualized as percentage-stacked bar charts (Figure 4A). Before air-frying, the major volatile compounds were acids (26.95%), esters (23.82%), ketones (20.72%), and sulfides (17.23%), followed by alcohols (6.52%), terpenes (2.80%), and aldehydes (1.97%). With prolonged frying, the relative contents of aldehydes and ketones continuously increased, reaching 7.78% and 34.45% at 20 min, respectively, indicating intensified lipid oxidation. In contrast, the relative abundances of esters and sulfides markedly decreased to 6.91% and 2.58%, respectively, at 20 min, suggesting their degradation or transformation during thermal reactions. Acids exhibited a rise-then-fall pattern, peaking at 15 min (39.64%), which was likely associated with fatty acid release and intermediate accumulation. The proportions of terpenes and alcohols fluctuated only slightly (variation < 4%). Overall, air-frying accelerated lipid oxidation and degradation, resulting in a notable increase in aldehydes and ketones, which play critical roles in the development of meaty and roasted aromas, whereas ketones may also help reduce the characteristic fishy odor of aquatic meat products (38–40).

Cluster analysis of 48 volatile compounds (Figure 4B) further revealed the dynamic evolution of flavor profiles. The heatmap, visualized in a red-to-blue gradient, represents high and low relative concentrations, respectively. The clustering results revealed that the 0 min and 5 min samples were closely related, retaining most of the raw meat's characteristic volatiles. The samples at 10 and 15 min clustered together, indicating similar VOC distributions and a more balanced aroma profile at this stage. In contrast, the 20 min sample formed a distinct cluster due to intense thermal reactions, characterized by caramelized and roasted notes. Specifically, esters such as ethyl 2-methylpropanoate D, ethyl 2-hydroxypropanoate, and ethyl acetate D, along with sulfide compounds such as diallyl disulfide (D and M), were abundant at early frying stages (0–5 min), contributing fruity and garlic-like aromas. Moreover, thermal degradation products and Maillard reaction products such as 2-heptanone (D and M) and 3-methylbutanal (D and M) accumulated significantly between 10 and 20 min, reaching peak concentrations at 20 min and imparting caramel-like aromas. Collectively, the samples fried for 10–15 min maintained their original freshness while generating moderate thermal reaction products, leading to a well-balanced aroma structure consistent with optimal sensory quality. However, excessive oxidation and pyrolysis at 20 min resulted in heavier, caramelized flavors.

### 3.5 Principal component analysis and orthogonal partial least squares discriminant analysis of giant salamander meat at different AF durations

**Figure 5:**
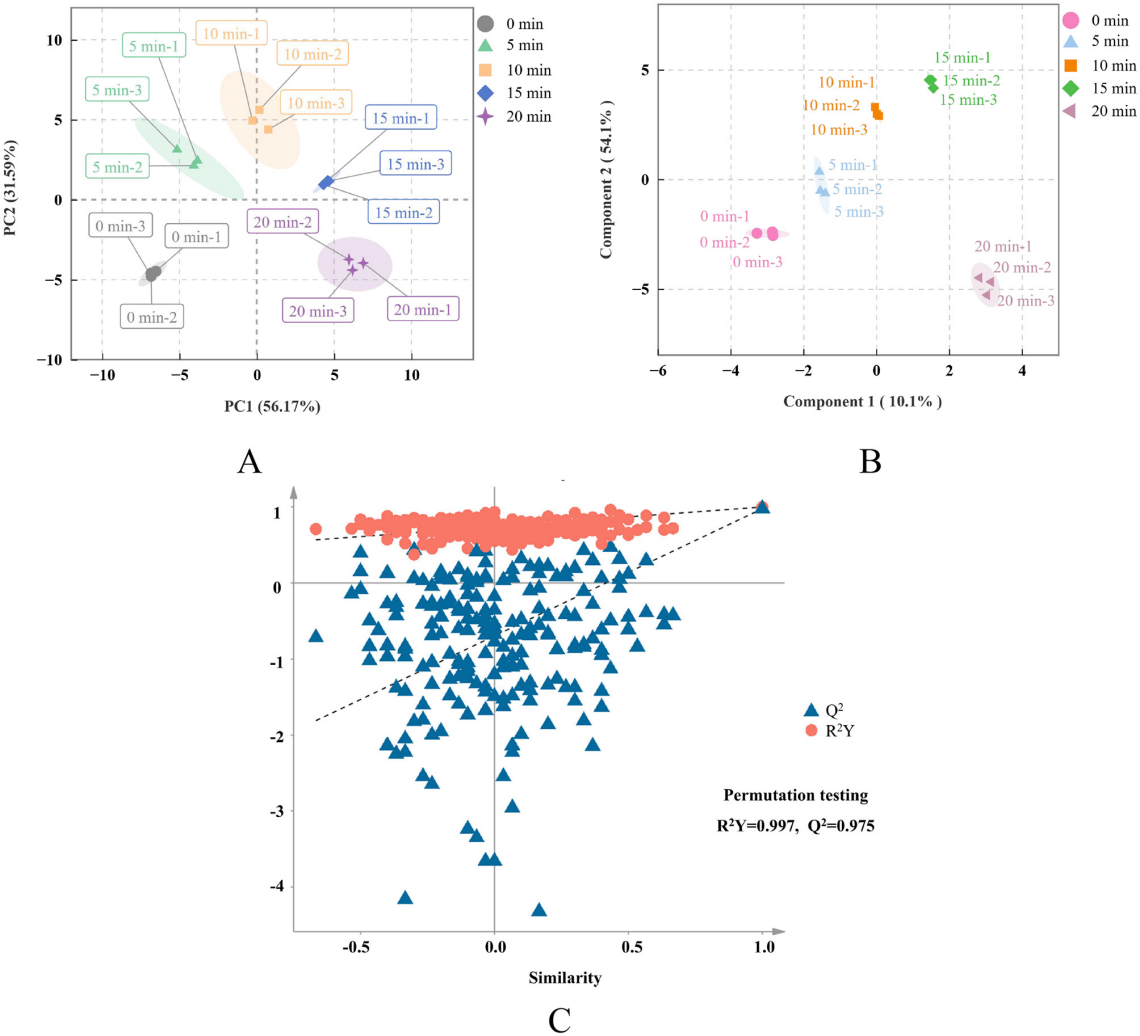
Multivariate statistical analysis of different frying durations points of giant salamander meat **(A)** PCA scores; **(B)** OPLS-DA model validation diagram; **(C)** permutation analysis test.

Principal component analysis (PCA) is a method for recognizing patterns without supervision that uses linear dimension reduction technology for multivariate data analysis (4, 41). Feng et al. (42) applied PCA to analyze VOCs when studying the consequences of vacuum belt drying on the flavor of honey powder at different temperatures and successfully distinguished samples at different drying temperatures, indicating the effectiveness of PCA in food flavor analysis. Therefore, this method can be used to determine the diversity in the variations in VOCs in giant salamander flesh instances with diverse durations of air frying. PCA involves the first two principal components of PC1 and PC2, and the contribution rate of each principal component represents its degree of interpretation of the overall data variance (43). Figure 5A shows that the first principal component (PC1) and the second principal component (PC2) contributed 56.17% and 31.59% of the diversification, respectively, with an overall contribution rate of 87.76%, indicating that PC1 and PC2 can effectively summarize the core change features of VOCs during frying. As shown in Figure 5A, five kinds of giant salamander meat samples with different frying durations formed a distribution in the PCA diagram, and the samples at each time point were closely clustered, indicating that the volatile components in the group were relatively stable. Moreover, the distance between samples at each time point was large, and there was no obvious overlap, indicating that the VOCs of giant salamander meat changed significantly with increasing air frying time.

To further screen the characteristic markers and evaluate the effects of different volatile substances on giant salamander meat samples at different frying durations, orthogonal partial least squares discriminant analysis (OPLS-DA) was also employed for in-depth analysis. Combined with orthogonal signal correction technology, this method can effectively remove irrelevant information and improve the discrimination ability and prediction accuracy of the model (44). OPLS-DA was performed on 48 volatile components to study the differential aroma compounds among samples with different frying durations. In Figure 5B, the fit index of the independent variable (*R*^2^_*x*_) was 0.985, the fit index of the dependent variable (*R*^2^_*y*_) was 0.997, and the forecasting capacity index (*Q*^2^) reached 0.975. *R*^2^ and *Q*^2^ were both >0.9, indicating that the model provides powerful elucidative power and sibylline robustness (45, 46) and can efficiently separate fried giant salamander meat samples in different periods on the basis of changes in volatile substances. To validate the reliability of the OPLS-DA model, 200 permutations were conducted. In each permutation, the sample grouping labels (*Y* variable) were randomly shuffled, and the OPLS-DA model was re-established to calculate the corresponding *R*^2^ and *Q*^2^ values. If the original model's *R*^2^ and *Q*^2^ values both significantly exceed the distribution of the permutation models and the *Q*^2^ intercept of the permutation regression line falls within the negative range, this indicates that the model possesses robust stability and predictive capability, with no evidence of overfitting (47). Figure 5C shows that the *R*^2^ regression line intersects the positive half-axis of the Y-axis, whereas the *Q*^2^ regression line intersects at the negative half-axis, further substantiating the model's reliability and favorable predictive performance.

### 3.6 Analysis of key flavor substances

#### 3.6.1 Identification of key flavor substances

**Figure 6:**
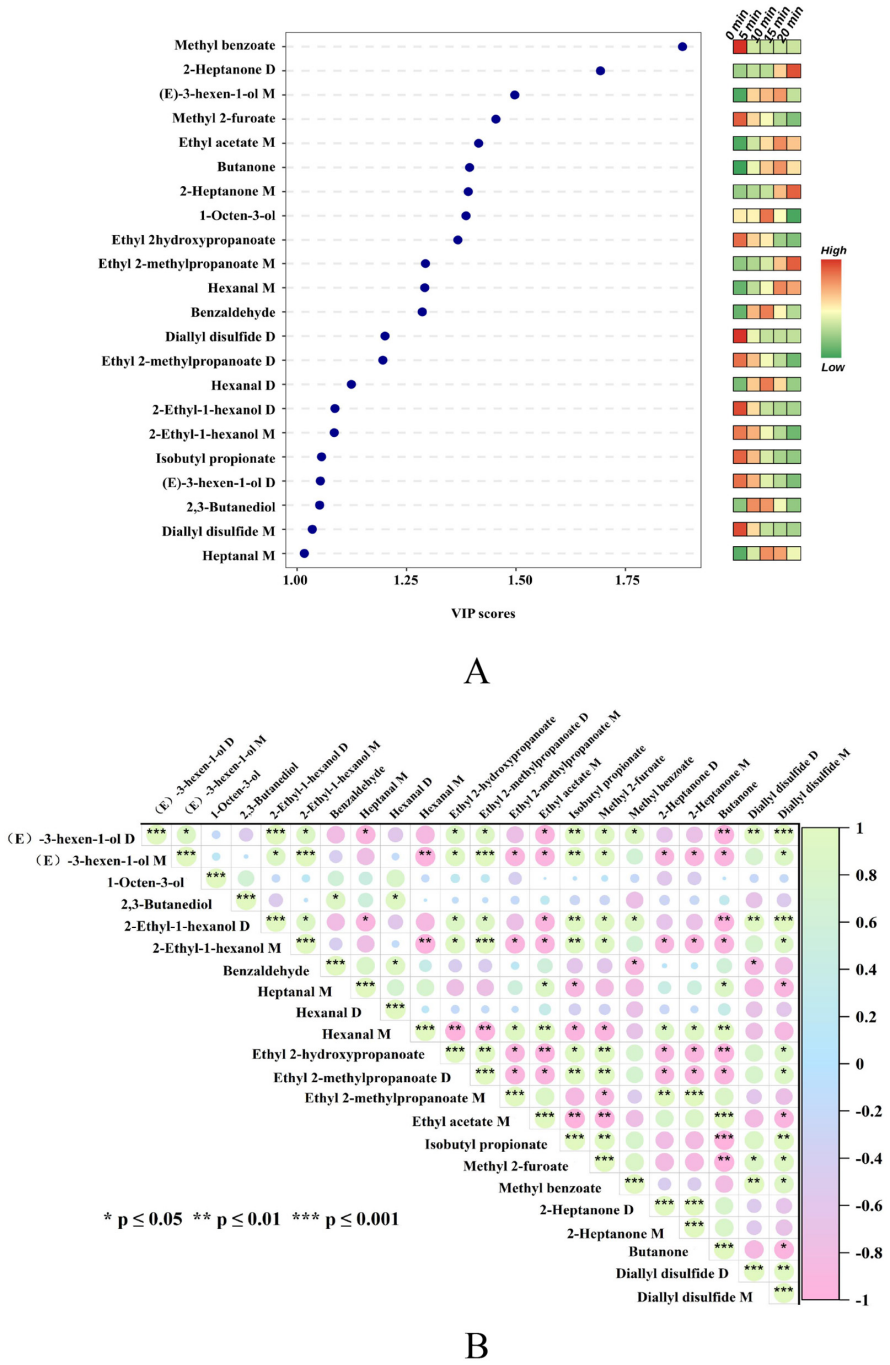
**(A)** VIP score of key volatile compounds identified by OPLS-DA; **(B)** Pearson correlation heatmap of VOCs with VIP 1.

On the basis of the GC–IMS fingerprint spectra, after the 48 volatile substances identified in the giant salamander meat samples from five different AF frying durations were visualized, and on the basis of the responsible OPLS-DA model, the variable importance in projection (VIP) values were evaluated, 22 essential flavor mixtures with VIP > 1 were screened out (Table 2). It can be alcohol ((E)-3-hexen-1-ol (D and M), 1-octen-3-ol, 2-ethyl-1-hexanol (D and M), 2,3-butanediol), an aldehyde (benzaldehyde, heptanal (M), hexanal (D and M)), an ester (methyl benzoate, methyl 2-furoate, ethyl acetate (M), ethyl 2-hydroxy propanoate, ethyl 2-methyl propanoate (D and M), isobutyl propionate), a ketone (butanone, 2-heptanone (D and M)), or a sulfide (diallyl disulfide (D and M)). In Figure 6A, each point represents a compound. The horizontal position shows its VIP score. The color bar on the right (from green to red) reflects the relative peak volume, with red indicating a higher content and green indicating a lower content. Generally, variables with a VIP > 1 are considered to contribute tremendously to the samples, and the higher the VIP value is, the more important its influence on flavor differences (48). Yan et al. (49), on the basis of GC–IMS data, successfully identified 20 potential aroma-active volatile compounds via the VIP value screening method (VIP > 1), and these marker compounds effectively distinguished Hulatang samples from different production regions, indicating that the VIP-based screening method has high reliability in the identification of aroma volatiles. The PCA results of these 22 substances (Supplementary Figure S1) showed that the total contribution ratio of the first two principal components collectively reached 92.92%, indicating that they can effectively distinguish giant salamander meat samples with different frying durations. These findings further prove that the 22 kinds of volatile organic compounds screened have important influences on the flavor characteristics of giant salamander meat throughout the frying procedure.

**Table 2:** Substances with a VIP >1 and their classification.

**Count**	**Classification**	**Index**	**CAS**	**VIP value**
1	Alcohols	(E)-3-hexen-1-ol D	C928972	1.054
2		(E)-3-hexen-1-ol M	C928972	1.497
3		1-Octen-3-ol	C3391864	1.386
4		2,3-Butanediol	C513859	1.052
5		2-Ethyl-1-hexanol D	C104767	1.086
6		2-Ethyl-1-hexanol M	C104767	1.085
7	Aldehydes	Benzaldehyde	C100527	1.286
8		Heptanal M	C111717	1.017
9		Hexanal D	C66251	1.124
10		Hexanal M	C66251	1.292
11	Esters	Ethyl 2-hydroxy propanoate	C97643	1.367
12		Ethyl 2-methyl propanoate D	C97621	1.196
13		Ethyl 2-methyl propanoate M	C97621	1.293
14		Ethyl acetate M	C141786	1.415
15		Isobutyl propionate	C540421	1.056
16		Methyl 2-furoate	C611132	1.454
17		Methyl benzoate	C93583	1.880
18	Ketones	2-Heptanone D	C110430	1.693
19		2-Heptanone M	C110430	1.391
20		Butanone	C78933	1.394
21	Sulfides	Diallyl disulfide D	C2179579	1.201
22		Diallyl disulfide M	C2179579	1.035

#### 3.6.2 Distribution characteristics of key flavor substances

**Figure 7:**
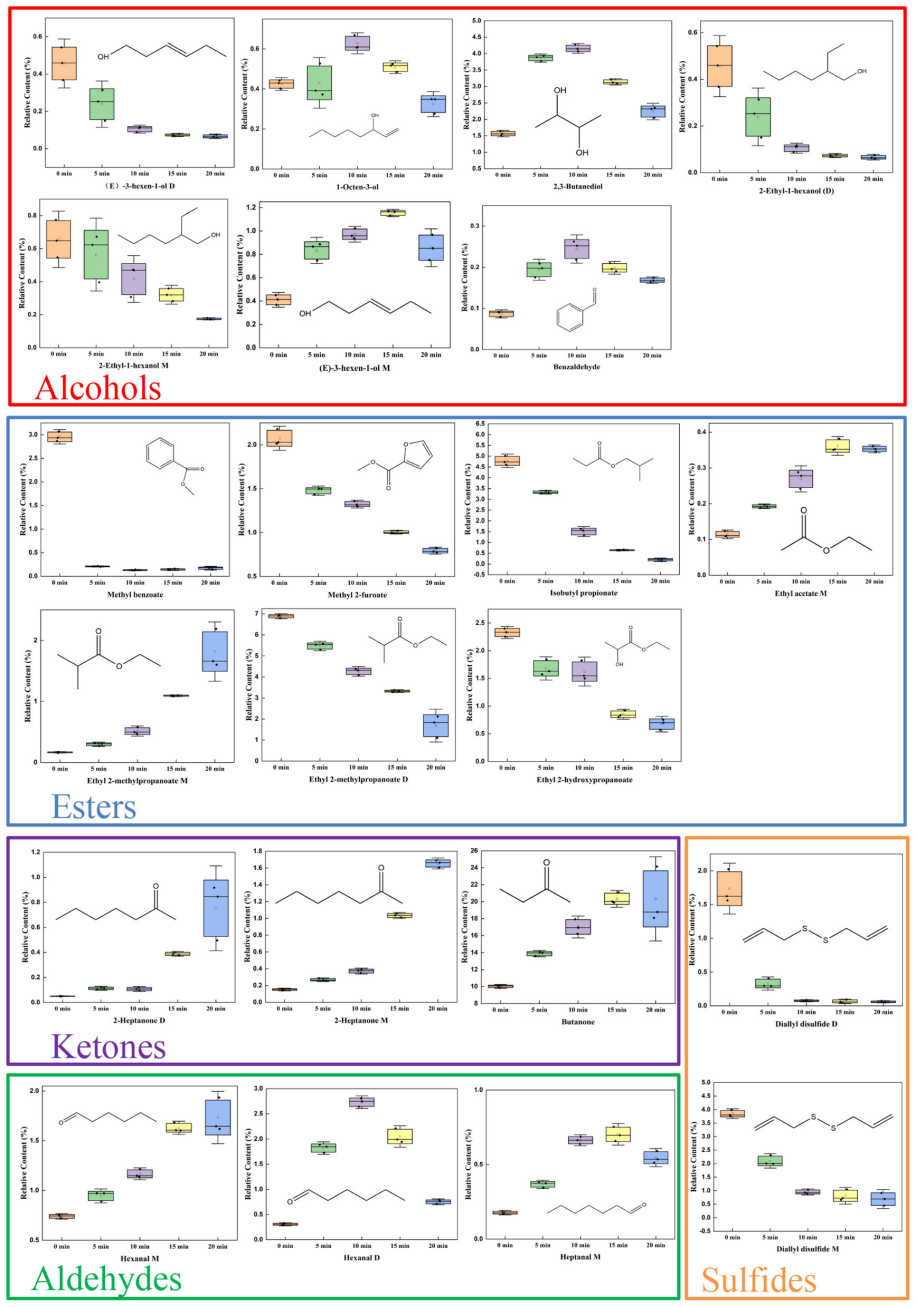
Boxplot of 22 volatile organic compounds (according to the selection criterion of VIP 1).

Box plots were generated for the 22 volatile compounds identified (Figure 7) to analyze the differential temporal response patterns exhibited by substances across different chemical categories during air frying. Within each box plot, the median line indicates relative abundance, whereas the box height reflects data dispersion. Higher or more concentrated abundances indicate the formation or accumulation characteristics of a compound during specific stages.

At 0 min, three alcohols ((E)-3-hexen-1-ol D, 2-ethyl-1-hexanol (D and M)), five esters (methyl benzoate, ethyl 2-hydroxypropanoate, isobutyl propionate, ethyl 2-methylpropanoate (D), methyl 2-furoate), and two sulfides (diallyl disulfide (D and M)) were present at significantly higher levels than in the other time periods, imparting the samples with fresh fruity and caramelized nutty sensory characteristics. Diallyl disulfide may originate from the garlic added during the pretreatment stage (50), whereas esters such as ethyl 2-hydroxypropanoate and methyl benzoate contribute a sweet and mellow aroma, building a multilayered odor foundation (51, 52). When the frying time reached 10 min, the concentrations of the lipid oxidation product 1-octen-3-ol and the Maillard reaction product benzaldehyde increased significantly, indicating that lipid oxidation had entered an active stage. 1-Octen-3-ol, derived from linoleic acid oxidation, imparts a mushroom-like aroma (53–55), whereas benzaldehyde is generated from phenylalanine degradation, presenting a nutty note (56). At this stage, the reactive carbonyl intermediates produced by lipid oxidation effectively promoted the Maillard reaction, driving the flavor system from initial accumulation toward a more complex baked aroma. By 15 min, the levels of aldehydes (heptanal M), ketones (butanone), and alcohols ((E)-3-hexen-1-ol M) continued to rise, increasing the baked attributes. Aldehydes, as low-threshold lipid oxidation products, contribute predominantly to meaty notes (57), whereas ketones and alcohols enhance flavor complexity with floral and fruity nuances (58, 59). At this point, the reaction system underwent a notable shift: lipid oxidation products interacted with protein amino groups through Maillard condensation and Strecker degradation to generate heterocyclic compounds with a roasted aroma, marking a key turning point in flavor evolution. At 20 min, hexanal M (green, fatty), 2-heptanone D and M (cheesy, nutty), and ethyl 2-methylpropanoate M (fruity) reached their peak levels, indicating that esterification (60) and lipid degradation (54) continued to intensify. The accumulation of 2-heptanone and hexanal confirms the dominant role of lipid degradation, whereas the thermal decomposition of Maillard intermediates and interactions among multiple components collectively generate nutty, roasted, and creamy aromas, forming a thermal reaction network consistent with that observed in other high-fat food systems (61–63).

The key flavor compounds mentioned above all presented VIP values > 1 in the OPLS-DA, and their dynamic trends were highly consistent with the clustering heatmap and sample appearance (Figure 1A). The samples fried for 10–15 min displayed a uniform light golden color, which corresponded well with the sensory descriptions of the mushroom and almond notes at 10 min and the roasted and floral aromas at 15 min (Figure 1E). Overall, these results indicate that 10–15 min is the optimal duration of air frying for giant salamander meat. During this period, the synergistic balance among lipid oxidation, Maillard reactions, and esterification produces the most desirable flavor profile.

#### 3.6.3 Correlation analysis of key flavor substances

Pearson correlation analysis was performed on the VOCs with a VIP > 1 (Figure 6B), where green represents positive correlations and pink indicates negative correlations. The results revealed significant associations among the compounds, suggesting a coupling effect among lipid oxidation, Maillard reactions, and esterification pathways. For example, alcohols (such as 2-ethyl-1-hexanol and (E)-3-hexen-1-ol) are strongly positively correlated with esters (such as ethyl 2-methylpropanoate), indicating that esterification between oxidized fatty acid intermediates and alcohols is a major pathway for ester formation (61). Additionally, the strong correlations among aldehydes, ketones, and alcohols, which are typical lipid oxidation products, highlight the dynamic balance between oxidation, reduction, and condensation reactions (62). Significant positive correlations were also observed across compound classes (*P* ≤ 0.01), such as between the ester ethyl 2-methylpropanoate M and the ketone 2-heptanone D, implying that carbonyl compounds derived from lipid oxidation can serve as substrates for secondary esterification or condensation, demonstrating the interdependence between lipid oxidation and the Maillard reaction pathways. Notably, 2-heptanone, which contributes a cheesy and nutty aroma, is a product of lipid degradation (63) and is closely related to meat flavor formation (64).

Negatively correlated compounds reveal competitive relationships among different reaction pathways. For example, (E)-3-hexen-1-ol D and M were negatively correlated with hexanal M, ethyl acetate M, and 2-heptanone D and M, indicating that as air frying progresses, lipid oxidation increasingly shifts toward aldehydes and ketones, depleting early alcohol intermediates. Hexanal, a typical product of linoleic acid β-oxidation, is associated with fatty off-flavors (65), whereas (E)-3-hexen-1-ol, an early oxidation intermediate, decreases due to continuous transformation or volatilization. This inverse relationship underscores the intrinsic mechanism underlying the flavor transition from fresh to roasted notes. Similarly, the negative correlations among butanone, isobutyl propionate, and methyl 2-furoate reflect competition for substrates between lipid cleavage and esterification under high-temperature conditions (59).

These mechanistic interactions collectively indicate that during air frying, lipid oxidation, Maillard reactions, and esterification alternately dominate and mutually constrain each other, forming a dynamic network that ultimately shapes the flavor profile. This finding provides a theoretical basis for process optimization, suggesting that precise control of the reaction intensity or adjustment of precursor availability could effectively suppress undesirable flavors, thereby enhancing the overall sensory quality and market acceptability of air-fried giant salamander meat.

### 3.7 Analysis of key flavor substances in AF giant salamander meat via the ROAV method

The relative odor activity value (ROAV) is widely employed in food flavor research to evaluate the contribution of volatile components to overall aroma, offering greater scientific rigor than simple concentration analysis. Compounds with ROAVs ≥ 1 are generally considered characteristic aroma compounds of a sample (66). This study employed the method of Liu et al. (67) to calculate ROAV, identifying 38 differentially volatile compounds. The results revealed that the ROAV of a total of 15 compounds was ≥1 (Table 3), and they were determined to be key flavor substances, including 6 aldehydes (octanal (D and M), hexanal (D and M), and 3-methylbutanal (D and M)), 3 esters (ethyl 2-methylpropanoate (D and M) and methyl benzoate), 2 kinds of acids (acetic acid (D and M)), 2 kinds of alcohols (1-octen-3-ol and isopentyl alcohol D), and 2 kinds of sulfides (diallyl disulfide (D and M)).

**Table 3:** Odor threshold and odor description of 15 volatile organic compounds with ROAV ≥ 1 in air at different frying duration points.

**Count**	**Classification**	**Index**	**CAS**	Odor Threshold value^a*^	Flavor description^b*^	**ROAV**				
						**0 min**	**5 min**	**10 min**	**15 min**	**20 min**
1	Acids	Acetic acid D	C64197	0.013	Acid, fruit, pungent, sour, vinegar	1.03	1.33	2.13	3.15	5.68
2		Acetic acid M	C64197	0.013	Acid, fruit, pungent, sour, vinegar	0.38	0.73	1.22	1.47	2.50
3	Alcohols	1-octen-3-ol	C3391864	0.0027	Mushroom	0.25	0.32	5.20	6.31	9.77
4		Isopentyl alcohol D	C123513	0.0061	Alcoholic, fusel, banana	1.26	1.90	0.40	1.17	2.93
5	Aldehydes	Octanal M	C124130	0.00088	Citrus, fatty, green, fried, pungent	0.71	2.62	1.51	4.11	13.5
6		Hexanal M	C66251	0.0014	Apple, fatty, fresh, green, fried	0.84	1.36	12.4	15.8	24.1
7		Hexanal D	C66251	0.0014	Apple, fatty, fresh, green, fried	0.35	2.61	0.14	0.16	0.28
8		3-methylbutanal M	C590863	0.00035	Nutty, caramel-like, malty aroma	1.33	1.78	2.14	4.85	14.1
9		3-methylbutanal D	C590863	0.00035	Nutty, caramel-like, malty aroma	1.48	1.10	8.60	15.4	32.3
10		Octanal D	C124130	0.00088	Citrus, fatty, green, fried, pungent	0.14	0.83	4.89	4.92	5.71
11	Esters	Ethyl 2-methylpropanoate D	C97621	0.00011	Fruity, sweet, ether-like aroma	100	100	100	100	100
12		Ethyl 2-methylpropanoate M	C97621	0.00011	Fruity, sweet, ether-like aroma	2.41	5.46	22.3	24.4	40.7
13		Methyl benzoate	C93583	0.0015	Floral, fruity, sweet aroma	3.15	0.28	2.24	4.82	12.7
14	Sulfides	Diallyl disulfide M	C2179579	0.0013	Garlic	4.73	3.25	0.26	0.37	0.86
15		Diallyl disulfide D	C2179579	0.0013	Garlic	2.13	0.51	5.42	5.21	3.78

a^*^: Odor threshold values (μg/m3, in air) were obtained from the Compendium of Olfactory Thresholds for Compounds (2nd ed.), Perception Thresholds for the Sense of Smell in Air.

b^*^: The odor descriptions are from https://mffi.sjtu.edu.cn/database/search?value=hexanoic+acid&keyword=all and https://www.femaflavor.org/flavor-library/hexanal.

From a chemical classification and olfactory perspective, ester compounds (e.g., ethyl 2-methylpropanoate and methyl benzoate) mainly originate from acid–alcohol esterification reactions, imparting fruity and sweet aromas that become more active at elevated temperatures (68, 69). Aldehydes (hexanal D and M, octanal D and M, and 3-methylbutanal D and M) are typical lipid oxidation products widely present in thermally processed meats, and contribute to the characteristic fatty and fried aroma of cooked products (58, 70). Alcohols (e.g., isopentyl alcohol D and 1-octen-3-ol) arise partly from lipid oxidation but may also form via amino acid degradation through the Maillard or Strecker pathway, providing mushroom and alcoholic notes (58, 70, 71). Sulfur compounds (diallyl disulfide D) are derived from the thermal degradation of sulfur-containing amino acids, producing distinctive garlic-like aromas (72). These findings indicate that the flavor development of air-fried giant salamander meat is not governed by a single reaction but rather by the synergistic interplay among lipid oxidation, Maillard reactions, esterification, and sulfur-compound cleavage.

Dynamic trend analysis revealed phased responses of different odor-active components during frying. The initial concentrations of compounds such as hexanal M, octanal M, and 1-octen-3-ol gradually increased, indicating that continuous lipid oxidation and amino acid thermal reactions resulted in the accumulation of aroma products (73, 74). Methyl benzoate maintained a consistently high ROAV throughout the process, which is consistent with the stability of its esterification pathway (58). Hexanal D sharply increased at 5 min, indicating a critical point for fatty acid oxidation acceleration (75, 76). Collectively, these representative VOCs form a complex flavor network through multiple interactive routes, lipid oxidation, the Maillard reaction, esterification, and sulfur compound degradation, defining the unique sensory profile of air-fried giant salamander meat.

Notably, among the 15 key compounds with ROAVs ≥ 1, eight (1-octen-3-ol, hexanal D and M, ethyl 2-methylpropanoate D and M, methyl benzoate, and diallyl disulfide D and M) simultaneously presented VIP values > 1, confirming that they are not only critical variables distinguishing sample groups but also major contributors to sensory perception. These compounds thus serve as key process control targets for optimizing aroma structure and product quality. Future research may further validate their quantitative flavor contributions through reconstitution or sensory quantitative analysis, thereby elucidating the interactive mechanisms between lipid oxidation and Maillard reactions in air-frying flavor formation with greater precision.

## 4 Conclusions

In conclusion, the shear force and a^*^ value of the meat slices increased notably (*P* < 0.05) as the duration of frying increased (0–20 min), whereas the processing yield and L^*^ value decreased prominently. The b^*^ value peaked at 15 min. HS-GC–IMS identified 48 volatile substances. The relative contents of ketones and acids are high. PCA effectively distinguished flavor differences (cumulative contribution of 87.76%), and OPLS-DA identified 22 key volatiles (VIP > 1). ROAV analysis revealed 15 key odor-active substances (ROAV ≥ 1), with 8 volatiles having the characteristics of VIP > 1 and being able to be used as core targets to distinguish the duration of air frying from flavor quality, which is worthy of further analysis and discussion. Considering the comprehensive chemical and physical characteristics, volatile components, and multivariate statistical analysis, the optimal processing time for air-frying giant salamander slices at 180 °C is 10–15 min. Future studies may incorporate multiple batches of raw materials, increase sample sizes, and apply multiomics approaches (such as metabolomics and lipidomics) to systematically investigate the correlations between volatile compounds and nutritional components, thereby elucidating the synergistic regulatory mechanisms of flavor and nutrition during the frying process.

## Data Availability

The original contributions presented in the study are included in the article/Supplementary material, further inquiries can be directed to the corresponding authors.
