# Supplementary figures and images for: Quality characteristics and volatile profiles of giant salamander (Andrias davidianus) meat during the air frying process
Source: Front Nutr. 2026 Jan 16;12:1649069. doi: 10.3389/fnut.2025.1649069 (PMC12858248; doi:10.3389/fnut.2025.1649069)

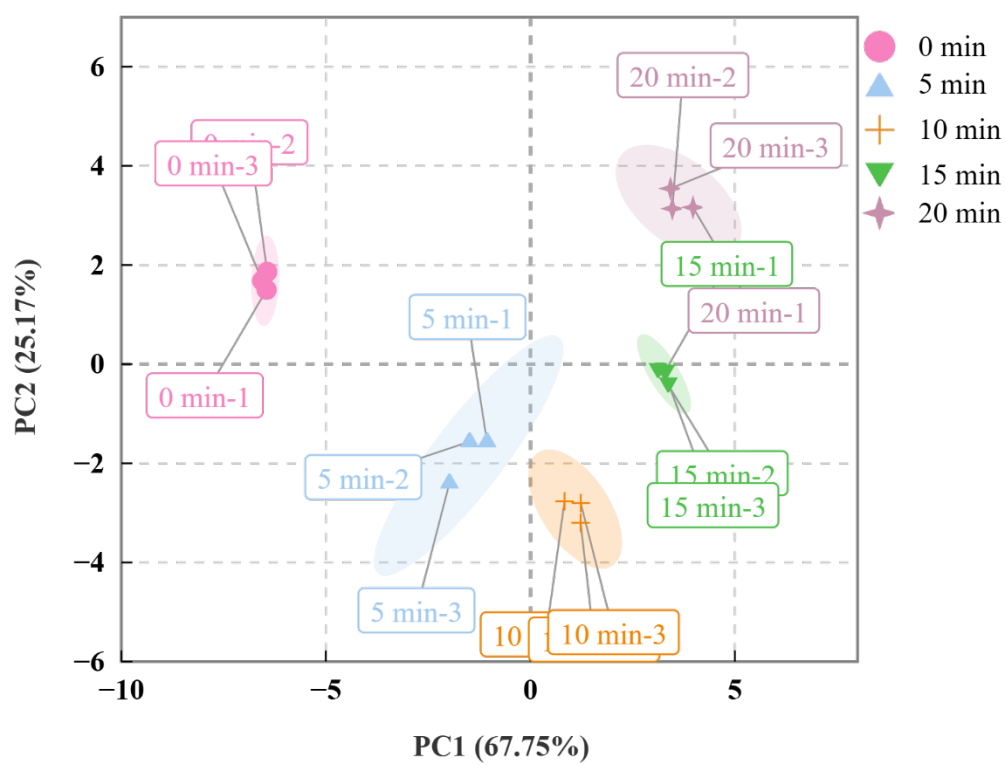

**Fig. S1:** PCA scores of 22 volatile organic compounds with VIP > 1

Supplement: Supplementary file 1 [file Image_1.pdf]
